# Stumbling blocks on the path to measles-free Nepal: impact of the COVID-19 pandemic

**DOI:** 10.1186/s41182-024-00576-6

**Published:** 2024-01-15

**Authors:** Chandan Kumar Thakur, Nitin Gupta, Nayanum Pokhrel, Samita Adhikari, Meghnath Dhimal, Pradip Gyanwali

**Affiliations:** 1 Research Section, Nepal Health Research Council, Ramshah Path, Kathmandu, Nepal; 2 Department of Infectious Diseases, Kasturba Medical College, Manipal, Manipal Academy of Higher Education, Manipal, India; 3 Department of Emergency, Nepal Mediciti Hospital, Lalitpur, Nepal

**Keywords:** COVID-19, Measles, Measles elimination, Measles outbreak, Routine immunization services, Vaccine preventable disease

## Abstract

Measles poses a significant global health threat, exacerbated by the COVID-19 pandemic. Despite the efficacy of two vaccine doses, under-5 mortality rates persist, with over 61 million delayed measles vaccinations worldwide. Nepal, striving to eliminate measles by 2023, faces a resurgence, attributing 1013 cases to inadequate vaccination and healthcare accessibility issues. Compounded by disruptions from the COVID-19 pandemic, the outbreak highlights the urgent need for vaccination promotion, improved healthcare access, and misinformation mitigation. This situation underscores the critical role of global collaboration and healthcare infrastructure investment to safeguard children's lives in Nepal and similar vulnerable regions.

Dear editor,

Measles is an acute viral respiratory illness caused by an RNA virus belonging to the *Paramyxoviridae* family [1]. It is highly contagious, and human transmission occurs through direct contact with respiratory secretions and aerosolized droplets from an infected person. The main symptoms include high-grade fever, cough, conjunctivitis, rash (exanthem and enanthem), and rhinitis [2]. The rash usually begins on the face and gradually spreads downward [3]. The contagious period starts four days before the appearance of the exanthem and ends 4 days after the rash has disappeared [3]. This disease has the potential to cause severe complications, particularly in young children (< 5 years), including pneumonia, otitis media, diarrhoea, encephalitis, myocarditis, and, in rare cases, death [3]. Even though two doses of vaccination effectively prevent the disease, this disease has been associated with high rates of under-5 mortality [4]. In this study, we discuss the hurdles to measles elimination in Nepal.

The World Health Organization (WHO) suggests 95% vaccination coverage with two doses of measles-containing vaccine (MCV) to attain herd immunity. The remaining 5% benefits from protection, as measles is unlikely to propagate in the vaccinated individuals. Herd immunity is crucial for preventing widespread outbreaks by reducing the number of susceptible individuals in the population and ensuring protection for those who cannot be vaccinated [4, 5]. There was steady progress in this century towards achieving the desired coverage rate before the projected global coverage for the first dose of the measles-containing vaccine dropped significantly, consequent to the ongoing COVID-19 pandemic [5]. This has been further accelerated by increased malnutrition in children during the COVID-19 pandemic, which corresponds with higher viral infection severity [1]. More than 61 million doses of measles-containing vaccine were either postponed or not administered [6]. Close to a quarter million children worldwide did not receive their first dose of the measles vaccine through routine immunization programs in 2021 [5]. The poor coverage has resulted in an upsurge in measles cases across the globe, especially in the South-East Asian region [7]. As of early July 2023, India and Pakistan in South Asia had the highest number of measles cases globally [6]. An upsurge in cases has been noted in other countries in the region, like Nepal. This has impacted the regional goal of WHO to eliminate measles by 2020 in the South-East Asian region [8].

Nepal is a mountainous, land-locked country in South Asia with more than 30 million people. In the early part of the century (2003), around 5000 measles cases were reported in a year. At the time, routine immunization included just a single dose of vaccine, and the coverage rate was only 75%. The coverage of vaccination since then has significantly increased across the years to 90%. This has resulted in a significant decline, with less than a hundred cases reported in 2017 [9, 10]. The goal of eradicating measles in 2020 was close to being achieved. However, after another upsurge of cases in Nepal and neighboring regions between 2019 and 2020 (Fig. 1), WHO has extended the timeline for achieving the eradication objective to 2023 [9]. According to recent reports, Nepal has experienced numerous measles cases with outbreaks and fatalities hindering its goal to eliminate measles by 2023, as targeted by the WHO South-East Asia Region. There were 1013 cases of measles in Western, Central, and Eastern Nepal reported from January to August 2023 (Fig. 1). Among these affected regions, Kailali district in Western Nepal Mahottari district in Central Nepal, and Sunsari district in Eastern Nepal reported the highest cases [11, 12]. The probable reasons for the outbreak were the lower vaccination coverage, poor vaccine delivery system, lack of cold chain maintenance, and incomplete vaccine doses. Additionally, vaccine hesitancy and changes in individual health-seeking behavior have been exaggerated by the COVID-19 pandemic. This is especially true in remote and disadvantaged communities. Underdeveloped routine immunization and poor community involvement have also contributed to the spread of the disease.

**Fig. 1:**
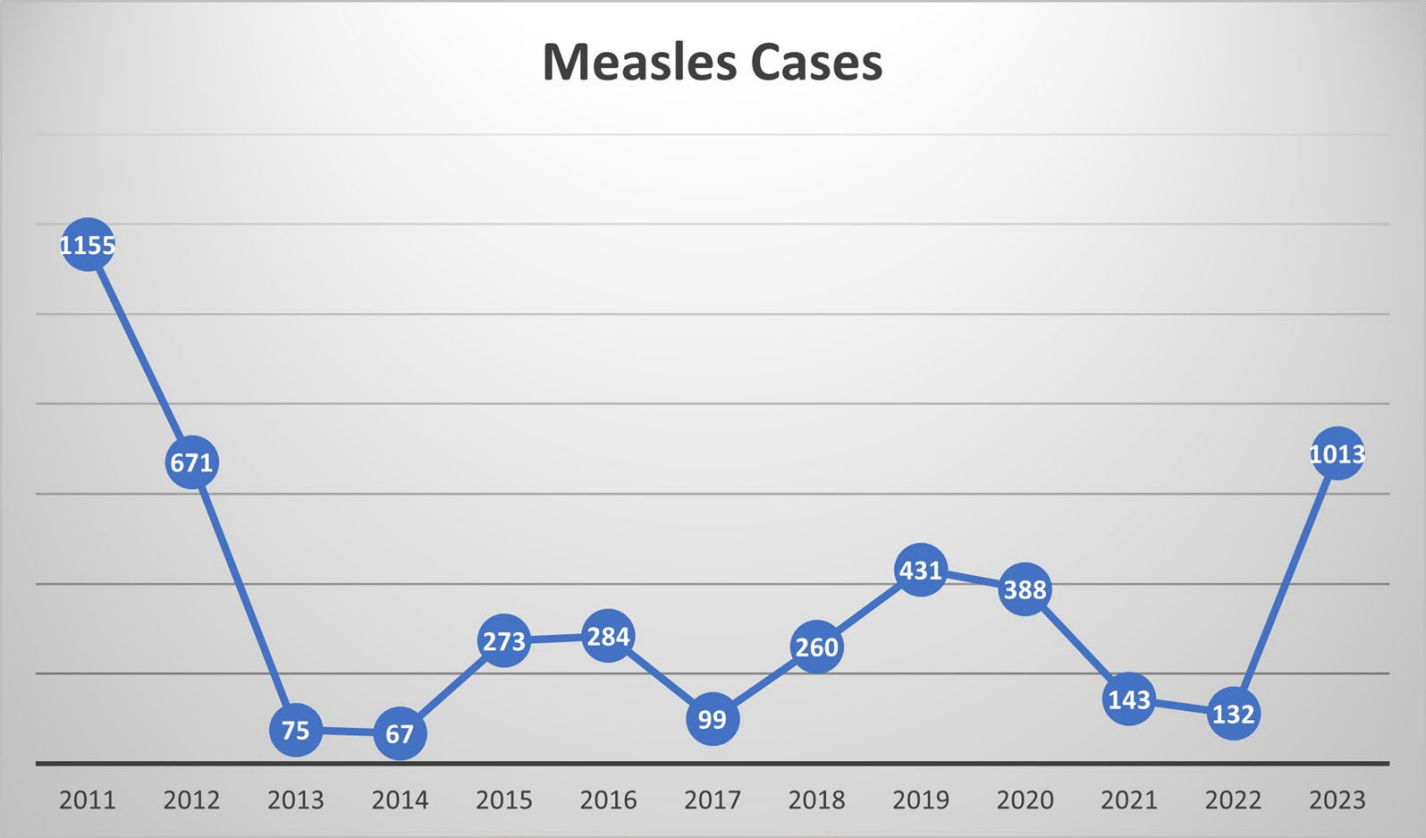
Number of cases of measles outbreaks from 2011 to 2023 in Nepal

Until July 2023, more than one million cases of COVID-19 have been diagnosed in Nepal, resulting in more than twelve thousand fatalities (ndrrma.gov.np). The pandemic and the control response have collateral effects, disrupting routine vaccination programs and disease surveillance systems. This might have not only led to an upsurge in the cases but also affected outbreak detection and response. This outbreak highlights the urgent need for increased efforts to promote vaccination and improve access, especially in remote areas. Additionally, efforts must be made to raise awareness about the importance of vaccination and address misinformation. As the number of COVID-19 cases has significantly decreased in Nepal, the manpower and infrastructure developed for COVID-19 immunization can be diverted to immunization against measles and other vaccine-preventable diseases. Furthermore, collaborative immunization strategies across borders and mandating the measles vaccine as a requirement for school entry can effective strategies to boost vaccination rates among families. There is also a need to devise strategies to help children catch up with the missed measles vaccination doses.

In conclusion, the recent measles outbreak in Nepal is a reminder of the importance of vaccination, monitoring malnutrition, administration of age-appropriate doses of vitamin A, strengthening measles surveillance, and mobilization with the overall need to invest in healthcare infrastructure. It serves as a wake-up call for the authorities to restore vaccination coverage and improve access to healthcare services. We must work together to ensure that every child in Nepal has unhindered access to life-saving vaccines and healthcare services.

## Data Availability

Not applicable.
